# The barley grain thioredoxin system – an update

**DOI:** 10.3389/fpls.2013.00151

**Published:** 2013-05-21

**Authors:** Per Hägglund, Olof Björnberg, Nicolas Navrot, Johanne Mørch Jensen, Kenji Maeda, Kristine Kirkensgaard, Azar Shahpiri, Abida Sultan, Jakob Bunkenborg, Frank Gubler, José Maria Barrero, Anette Henriksen, Christine Finnie, Birte Svensson

**Affiliations:** ^1^Enzyme and Protein Chemistry, Department of Systems Biology, Technical University of DenmarkKongens Lyngby, Denmark; ^2^Department of Agricultural Biotechnology, College of Agriculture, Isfahan University of TechnologyIsfahan, Iran; ^3^Center of Experimental Bioinformatics, Department of Biochemistry and Molecular Biology, University of Southern DenmarkOdense, Denmark; ^4^Commonwealth Scientific and Industrial Research Organisation Plant IndustryCanberra, ACT, Australia; ^5^The Protein Chemistry Group, Carlsberg LaboratoryCopenhagen, Denmark

**Keywords:** thioredoxin, disulfide bond, redox regulation, NADPH-dependent thioredoxin reductase, cereal proteomics

## Abstract

Thioredoxin (Trx) reduces disulfide bonds and play numerous important functions in plants. In cereal seeds, cytosolic h-type Trx facilitates the release of energy reserves during the germination process and is recycled by NADPH-dependent Trx reductase. This review presents a summary of the research conducted during the last 10 years to elucidate the structure and function of the barley seed Trx system at the molecular level combined with proteomic approaches to identify target proteins.

## INTRODUCTION

Thioredoxins (Trx) are ubiquitous small redox-active proteins that act as electron donors in various metabolic pathways, regulate enzymatic activities, and maintain the cellular environment in a reduced state (Arnér and Holmgren, 2000). Trx contains a WC[G/P]PC active site motif and reduces disulfide bonds in target proteins through thiol-disulfide exchange reactions. Oxidized Trx is recycled by the NADPH-dependent dimeric flavoprotein Trx reductase (NTR) or the chloroplastic iron-sulfur cluster proteins ferredoxin and ferredoxin–Trx reductase coupled to the photosynthetic apparatus (Jacquot et al., 2009). In NTR, reducing equivalents are first transferred from NADPH to a tightly bound FAD in the so-called flavin reducing (FR) conformation. Then, the enzyme undergoes a large conformational change and the redox active CXXC motif in NTR is positioned close to the reduced cofactor (FADH2) to allow electron transfer and disulfide reduction in the flavin oxidizing (FO) conformation. To complete a catalytic cycle the enzyme returns to the FR conformation and the active site WC[G/P]PC in Trx is reduced concomitant with oxidation of the NTR CXXC motif.

Plant Trx play key roles in regulation of processes such as photosynthesis, flowering, immunity, and seed germination (Buchanan and Balmer, 2005). In comparison to other organisms, plants contain a remarkable diversity of Trx classified into groups based on sequence similarity and showing different subcellular location (Gelhaye et al., 2005). The mainly cytosolic h-type Trx is reduced by NTR and is proposed to facilitate the germination and post-germination processes of cereal grains by (i) inactivating small proteinaceous inhibitors of proteolytic and amylolytic enzymes, (ii) activating hydrolytic enzymes such as thiocalsin and pullulanase, and (iii) enhancing the solubility of storage proteins (Kobrehel et al., 1991, 1992; Besse et al., 1996). Overexpression of Trx h in cereal seeds thus correlates with an accelerated germination rate (Wong et al., 2002; Li et al., 2009).

This review gives an overview of the Trx system of germinating barley seed consisting of two Trx h isoforms (HvTrxh1 and HvTrxh2) that are recycled by two NTRs (HvNTR1 and HvNTR2). Proteomic approaches used to identify barley Trx-target proteins and the molecular features of protein–protein interactions in the barley Trx system are described.

## GENE REGULATION AND PROTEIN APPEARANCE PROFILES

Indications that the HvTrxh1 and HvTrxh2 may have distinct roles in seeds came initially from proteome analysis of mature barley grains, where the two gene products were observed for the first time and showed differences in appearance profiles (Maeda et al., 2003). HvTrxh1 appeared in two 2D gel spots with similar intensities in the starchy endosperm, aleurone layer and embryo, whereas HvTrxh2 in a single spot was predominant in the embryo. During germination of the seeds, the amount of HvTrxh2 and one HvTrxh1 spot decreased in intensity (Maeda et al., 2003; Bønsager et al., 2007), however, the second HvTrxh1-containing spot remained at similar intensity in the embryo of germinated seeds (Maeda et al., 2003). In isolated aleurone layers incubated with the plant hormones gibberellic acid (GA) or abscisic acid (ABA), a single HvTrxh1-containing spot was identified by Western blotting and mass spectrometry (Shahpiri et al., 2008). Semi-quantitative RT-PCR of seed tissues showed that genes encoding both HvTrxh1 and HvTrxh2 were expressed at relatively constant levels in germinating embryos and in isolated aleurone layers with or without hormone treatment (Shahpiri et al., 2008). This was subsequently confirmed by quantitative PCR (Kirkensgaard, 2011). The clearly observed differences in HvTrxh protein profiles therefore seem not to be due to regulation of transcription and probably occur at the post-translational level.

Transcripts encoding both HvNTR1 and HvNTR2 were detected in embryos isolated from mature grains and increased up to 72 h after imbibition (Shahpiri et al., 2008). Transcripts were also detected for both genes in isolated aleurone layers. HvNTR2 transcript was present at similar levels in embryo and aleurone layer whereas HvNTR1 transcripts were much less abundant in the embryo than in the aleurone layer. The HvNTR2 transcript level was reduced in aleurone layers treated with GA for up to 18 h (Shahpiri et al., 2008). Quantitative PCR (Kirkensgaard, 2011) showed a slight downregulation of HvNTR1, and a more than twofold upregulation of HvNTR2 by ≥100 nM GA after 24 h. The expression level of HvNTR2 was therefore confirmed to be 10–40 times higher than HvNTR1, suggesting that HvNTR2 is the most important isoform in aleurone layers subjected to GA.

The levels for both HvTrxh transcripts were around five times higher than even the highest level of HvNTR2. Overall, the data suggest that the activity level of the NTR/Trx system in barley grain tissues is determined by transcriptional regulation of NTR genes, coupled with post-translational regulation of HvTrxh protein levels. In this context it is relevant to point out that barley microarray analysis has shown that loss of dormancy leads to increased expression of HvNTR1, HvNTR2, and HvTrxh1 in embryos of imbibed grains (Barrero et al., 2009; Kirkensgaard, 2011).

## STRUCTURAL AND CATALYTIC PROPERTIES

HvTrxh1 and HvTrxh2 show 51% sequence identity and similar biophysical characteristics. The redox potentials (*E*°′) of both proteins was determined to be -270 mV in a fluorometric assay using *Escherichia coli* Trx as a reference and the pK_a_ of the nucleophilic active site thiol (CGPC) in both HvTrxh1 and HvTrxh2 were determined to be 7.6 by iodoacetamide (IAM) alkylation kinetics (Maeda et al., 2010). Nevertheless, HvTrxh1 displays slightly higher thiol reactivity and higher affinity for the model substrate insulin, possibly due to subtle differences in the local environment surrounding the active site. The three-dimensional crystal structures of HvTrxh1 and HvTrxh2 were determined to 1.7 and 2.0 Å resolution, respectively (Maeda et al., 2008). Both proteins display the overall fold conserved among Trx from different species with a central five-stranded β-sheet surrounded by four α-helices in a βαβαβαββα topology. Comparison of the structures of HvTrxh2 determined in oxidized and partially reduced states does not suggest major redox-dependent changes in the active site area with the exception of the side chain conformations of the redox-active cysteines (Maeda et al., 2008). Dimers of HvTrxh1 are formed in the crystal lattice and the interface is stabilized by three backbone–backbone hydrogen bonds in a pattern that resembles the intermolecular contacts observed in Trx-target complexes (see below).

The structure of HvNTR2 was solved to 2.6 Å resolution by X-ray crystallography (Kirkensgaard et al., 2009). As expected, this first example of a monocotyledonous plant NTR structure reveals a dimeric protein in which each monomer is composed of FAD- and NADPH-binding domains. HvNTR2 share overall similarity to the structures of AtNTR-B from *Arabidopsis thaliana* and other low-molecular-weight NTRs (Jacquot et al., 2009). However, the relative position of the FAD and the NADPH domains is not the same. Compared to AtNTR-B the NADPH domain in HvNTR2 is rotated by 25° and bent by a 38% closure relative to the FAD domain. The structure may thus represent an intermediate between the FO and the FR conformations.

Given that both HvTrxh1-,HvTrxh2-,HvNTR1-, and HvNTR2-encoding genes are expressed to some extent in all grain tissues, it was relevant to determine whether the proteins could function interchangeably. This was shown to be the case, with minor variations in catalytic efficiency (Shahpiri et al., 2008). Importantly, the activity of the system was confirmed at the relatively low pH expected in the starchy endosperm of germinating grains (Shahpiri et al., 2008).

## PROTEOMIC APPROACHES FOR IDENTIFICATION OF BARLEY Trx h TARGET PROTEINS

**FIGURE 1:**
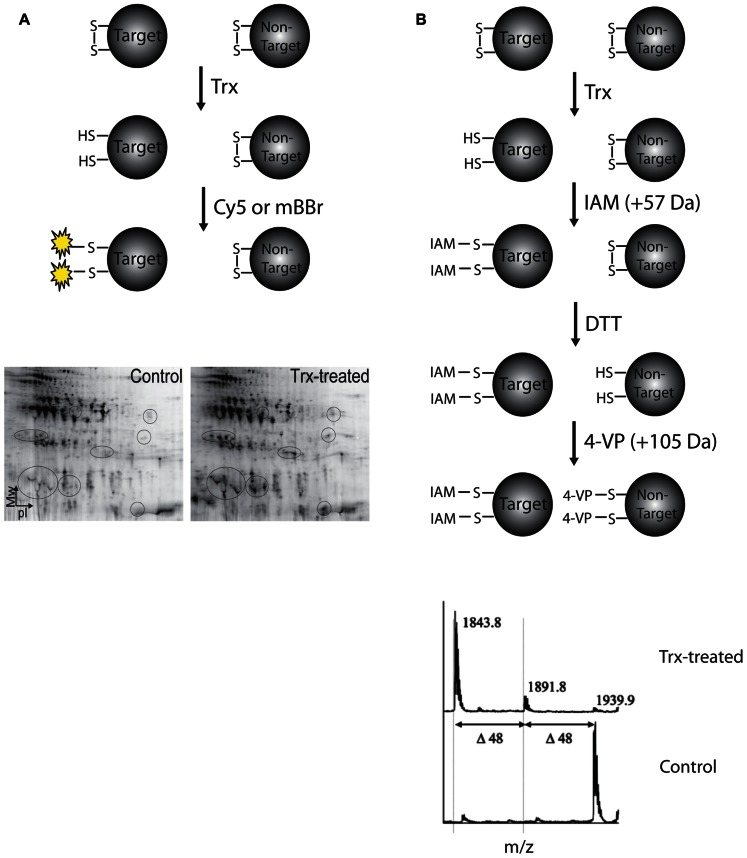
**Procedures for identification of Trx h targets based on protein separation by two-dimensional gel electrophoresis**. **(A)** Identification of target proteins using fluorescent dyes such as MBBr or Cy5. Fluorescence-scanned 2D-gels of mature barley seed proteins treated +/- Trx. The appearance of additional Cy5-labeled spots in the Trx-treated sample is indicated with circles. **(B)** Differential labeling procedure using iodoacetamide (IAM) and 4-vinylpyridine (4-VP) for identification of Trx h-reducible protein disulfides (top). Sections of matrix-assisted laser desorption/ionization time-of-flight (MALDI-TOF) mass spectra for the tryptic peptide ^141^LMSCGDWCQDLGVFR^155^ (M+H = 1729.75 Da with reduced [-SH] cysteine residues) from α-amylase/subtilisin inhibitor BASI containing the Cys144–Cys148 disulfide exposed to differential thiol labeling (bottom). In control (the lower spectrum), a major peak at *m*/*z* 1939.9 is observed corresponding to the 4-VP pyridylethylated form of Cys144 and Cys148 (mass increase 2 × 105 Da). When treated with Trx (the upper spectrum), the base peak is shifted by 96 Da (2 × 105 Da - 2 × 57 Da) and appears at *m*/*z* 1843.8 instead corresponding to the IAM carbamidomethylated form of Cys144 and Cys148 (mass increase 2 × 57 Da), showing that these cysteines in BASI are involved in a Trx h-reducible disulfide. The minor peak at 1891.8 Da corresponds to a peptide containing one pyridylethylated and one carbamidomethylated cysteine residue, respectively (1 × 105 Da + 1 × 57 Da).

Target proteins of barley Trx h have been identified by different proteomic approaches applied to extracts of barley grain tissue. Briefly, protein extracts are incubated in the presence of recombinant Trx h and reduced thiols are labeled with specific reagents that are either visualized after separation by two-dimensional gel electrophoresis (2DE) or detected by a characteristic mass/charge ratio in a mass spectrometer (**Figure 1**). In the first proteomic investigation of Trx-target proteins in germinating barley embryo, Marx et al. (2003) used monobromobimane (mBBr) for visualization of proteins from barley embryo extracts reduced by Trx and separated by 2DE. Subsequently 16 target proteins including several α-amylase/trypsin inhibitors, chitinases, and cyclophilin, were detected in extracts of mature and germinating seeds using the more sensitive fluorescent cyanine dye Cy5 (Maeda et al., 2004). To detect specific disulfide targets in proteins separated by 2DE, a differential thiol-labeling procedure was developed and applied to proteins from mature barley seed extract (Maeda et al., 2005). Briefly, cysteines from disulfides reduced by Trx h were blocked with IAM followed by full reduction by dithiothreitol (DTT) and alkylation by 4-vinylpyridine (4-VP). Following trypsin digestion, peptides containing cysteines reacted with IAM and 4-VP were distinguished by mass shifts of 57 and 105 Da, respectively (**Figure 1B**). Thus, nine disulfides mainly originating from α-amylase/protease inhibitors were identified as Trx substrates (Maeda et al., 2005).

**FIGURE 2:**
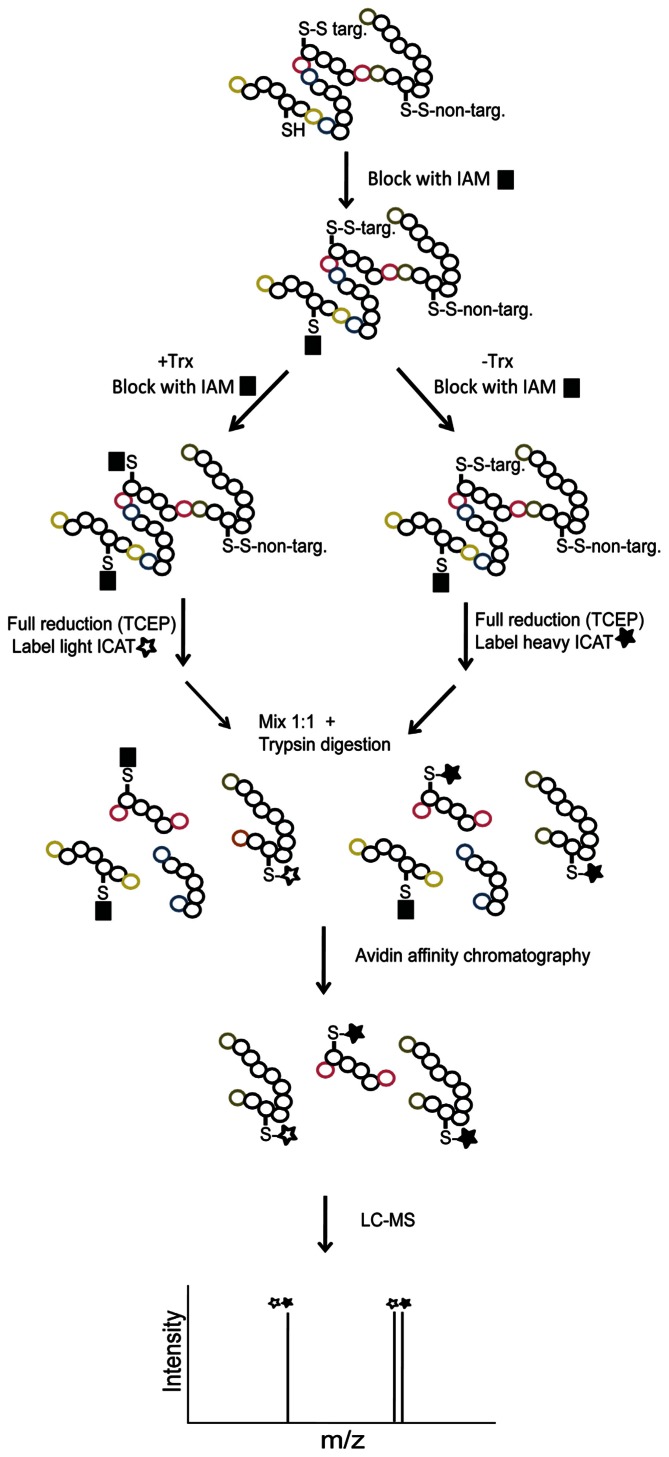
**Workflow for Trx h target identification using ICAT labeling and LC–MS/MS**. Free reduced cysteine thiols (SH) are first blocked with IAM. Samples are incubated +/- Trx for reduction of target disulfides (S–S target) and treated with IAM to block free thiols released by Trx. Remaining oxidized thiols (S–S non-target) are then reduced using TCEP and free thiols are labeled with ICAT_L_/ICAT_H_. Following tryptic digestion, the ICAT-labeled peptides are enriched by avidin affinity chromatography and analyzed by LC–MS/MS for peptide identification and determination of ICAT_H_/ICAT_L_ labeling ratios. ICAT_H_/ICAT_L_ peptide ratios of 1 are expected for non-target disulfide bonds and ratios >1 are expected for peptides containing cysteines from disulfide bonds reduced by Trx. The C- and N-terminal ends are labeled with colors to help tracking the origin of the tryptic peptides.

A gel-free proteomics approach for Trx-target identification was developed based on isotope-coded affinity tags (ICAT) labeling followed by liquid chromatography–mass spectrometry (LC–MS) analysis (Hägglund et al., 2008). The ICAT reagents contain a thiol-reactive IAM group and isotope-coded linkers in “light” (ICAT_L_) and “heavy” (ICAT_H_) forms labeled with nine ^12^C and ^13^C carbon atoms, respectively. Since the only difference between ICAT_L_ and ICAT_H_ is the number of ^12^C/^13^C atoms, it is possible to quantify the labeling ratio in a mass spectrometer. Furthermore, the ICAT reagents contain a biotin tag for selective enrichment of labeled species. In order to adapt ICAT labeling for relative quantification of Trx-target disulfide reduction, samples were first incubated in the presence or absence of Trx followed by IAM quenching. Then remaining thiols were chemically reduced with tris(2-carboxyethyl)phosphine (TCEP) and labeled with ICAT_L_ and ICAT_H_, respectively (**Figure 2**). The two samples were then mixed and digested by trypsin. ICAT-labeled peptides were isolated by avidin affinity chromatography and analyzed by LC–MS/MS to identify peptides and quantify ICAT_H_/ICAT_L_ ratios. Using this workflow ICAT_H_/ICAT_L_ peptide ratios of 1 are expected for non-target disulfide bonds and ratios >1 are expected for peptides containing cysteines from disulfide bonds reduced by Trx. The ICAT approach was applied to extracts of dissected embryo and proteins released from aleurone layers resulting in the identification of more than 100 putative targets (Hägglund et al., 2008, 2010). The most extensively reduced target from barley embryo was dehydroascorbate reductase suggesting a possible link between the Trx system and the ascorbate/glutathione cycle.

## MOLECULAR DETAILS OF TARGET RECOGNITION BY Trx h

The barley α-amylase/subtilisin inhibitor (BASI) contains two disulfide bonds located in the vicinity of the interfaces in contact with α-amylase and subtilisin, respectively. The disulfide close to the α-amylase surface was identified as a Trx h target by differential thiol labeling (Maeda et al., 2005) as outlined above (**Figure 1B**). To get further insight into the mechanism of Trx h-mediated reduction of this target disulfide, a complex of HvTrxh2 and BASI stabilized by an intermolecular disulfide bond was formed using single-cysteine mutants (HvTrxh2 C49S and BASI C144S). The structure of HvTrxh2-S-S-BASI at a resolution of 2.3 Å was determined by X-ray crystallography (Maeda et al., 2006). The complex is stabilized by numerous van der Waals contacts and three intermolecular hydrogen bonds involving the backbone of HvTrxh2 M88 and A106. This pattern of hydrogen bonds appears to be conserved among related thiol oxidoreductases in the Trx fold superfamily. To probe the importance of these hydrogen bonds, two HvTrxh2 variants M88P and A106P lacking the ability to form amide backbone hydrogen bond were constructed and assayed for activity toward target proteins and NTR. Enzyme kinetics indeed demonstrated that backbone hydrogen bonding involving A106 is important for interactions with BASI but appears not to affect reactivity with NTR (Björnberg et al., 2012). The M88P mutant was severely affected in terms of thiol reactivity and the role of M88 in target recognition could therefore not be conclusively demonstrated. Noticeably, an electrostatic contact between HvTrxh2 and BASI was engineered through a HvTrxh2 E86R mutation, which resulted in a threefold increase in disulfide reductase activity toward BASI (Björnberg et al., 2012).

Barley limit dextrinase inhibitor (LDI) contains nine cysteine residues forming four intramolecular disulfide bonds and a mixed disulfide with glutathione. Experiments with recombinant LDI *in vitro* revealed preferential reduction of the glutathionylated residue as well as complete disulfide reduction mediated by HvTrxh1 and HvTrxh2 (Jensen et al., 2012). Disulfide reduction correlates with loss of inhibitory activity proposed to occur due to conformational destabilization of reduced LDI.

## PROSPECTS FOR INDUSTRIAL APPLICATIONS

Cereal crops are highly valuable for the nutrition of livestock due to the high percentage of carbohydrates, storage proteins, starch, fatty acids, and vitamins. Barley grains contain relatively low amounts of protein compared to other crops, for example legumes. Following germination, the protein reserves are mobilized by proteases released from the aleurone layer as well as by the pre-formed proteases already present in the endosperm. Overexpression of Trx in transgenic barley endosperm resulted in an increase in protein solubility (Wong et al., 2002). Trx was also used for modification of solubility of proteins in wheat endosperm (Wong et al., 2004; Li et al., 2009). Proteome analysis of a barley grain-based “liquid feed” system showed that incubation with a functional NTR/Trx system increased the solubility of known Trx-target proteins (Sultan, A., Bjerg Christensen, J., Damgaard Poulsen, H., Svensson, B., and Finnie, C., unpublished results). Facilitating mobilization of the protein and starch reserves or increasing protein solubility through the application of the Trx system could be of great interest for the improvement of digestibility of animal feed.

## CONCLUSIONS AND PERSPECTIVES

The Trx system is of paramount importance for thiol redox control in germinating barley seeds and has a potential in industrial applications. Although the structural properties of the barley Trx system has been studied in great detail and a wide array of Trx h target have been identified, the differences in functional importance between the two pairs of Trx and NTR gene products described herein remains elusive. It would therefore be of interest to develop transgenic seeds expressing different combinations of HvNTR1/HvNTR2/HvTrxh1/HvTrxh2 and compare their performance in grain germination assays.

## Conflict of Interest Statement

The authors declare that the research was conducted in the absence of any commercial or financial relationships that could be construed as a potential conflict of interest.
